# Non-consensual condom removal, reported by patients at a sexual health clinic in Melbourne, Australia

**DOI:** 10.1371/journal.pone.0209779

**Published:** 2018-12-26

**Authors:** Rosie L. Latimer, Lenka A. Vodstrcil, Christopher K. Fairley, Vincent J. Cornelisse, Eric P. F. Chow, Tim R. H. Read, Catriona S. Bradshaw

**Affiliations:** 1 Central Clinical School, Monash University, Melbourne, Australia; 2 Melbourne Sexual Health Centre, Alfred Health, Melbourne, Australia; China Medical University, CHINA

## Abstract

**Background:**

Non-consensual removal of condoms, colloquially referred to as ‘stealthing’, is the removal of a condom during sex by a sexual partner when consent has been given for sex with a condom only.

**Methods:**

We conducted a cross-sectional survey to determine how commonly women and men who have sex with men (MSM) attending Melbourne Sexual Health Centre had experienced stealthing, and analysed situational factors associated with the event. Responses were linked to demographic information extracted from patient files.

**Results:**

1189 of 2883 women (41.2%), and 1063 of 3439 MSM (30.9%) attending the clinic during the study period completed the survey. Thirty-two percent of women (95% CI: 29%,35%) and 19% of MSM (95% CI: 17%,22%) reported having ever experienced stealthing. Women who had been stealthed were more likely to be a current sex worker (Adjusted Odds Ratio [AOR] 2.87, 95% CI: 2.01,4.11, p <0.001). MSM who had experienced stealthing were more likely to report anxiety or depression (AOR 2.13, 95% CI: 1.25,3.60, p = 0.005). Both female and male participants who had experienced stealthing were three times less likely to consider it to be sexual assault than participants who had not experienced it (OR 0.29, 95% CI: 0.22,0.4 and OR 0.31, 95% CI: 0.21,0.45 respectively).

**Conclusions:**

A high proportion of women and MSM attending a sexual health service reported having experienced stealthing. While further investigation is needed into the prevalence of stealthing in the general community, clinicians should be aware of this practice and consider integrating this question into their sexual health consultation. Understanding situational factors would assist in the development of preventive strategies, particularly female sex workers and MSM.

## Introduction

Non-consensual removal of condoms, colloquially referred to as ‘stealthing’[1] or ‘stealth-breeding’ [2], refers to the practice of a sexual partner covertly removing a condom, when consent has been given for condom protected sex only [1]. Condoms are used as a primary preventative method of protecting against sexually transmitted infections (STI), human immunodeficiency virus (HIV) and pregnancy, being 80 to 98.6% effective [3–5]. Stealthing may result in the transmission of STIs, HIV, or unintended pregnancy, and could have significant personal and public health implications.

Studies of undergraduate students have found consent for sexual intercourse to be mostly communicated through non-verbal means [6, 7], with consent for sexual intercourse often implied in the process of asking for or applying a condom [6]. Brodsky has argued that condom removal without mutual agreement violates consent to sex [1].

In young adult heterosexual relations, it is common for male partners to engage in condom resistance tactics [8]. Several studies have identified stealthing as a method of birth control sabotage [9, 10], as well as a means of intentional HIV transmission [11]. Anecdotal research by Brodsky focusing on heterosexual and heteronormative relations, and theoretical research by Brennan focusing on condom-less sex between men, argue these are not the primary motivators for this act [1, 2].

In spite of public interest in stealthing, there are no scientific articles that investigate how common it is, who is most at risk, and the outcomes for those who report being stealthed. We aimed to investigate the proportion of sexual health centre patients reporting nonconsensual removal of condoms: 1) among heterosexual women and 2) among men who have sex with men, as well as associated risk factors. For the purpose of this study, ‘stealthing’ was defined as condom removal without consent, where consent to sex was conditional upon use of a condom.

## Methods

### Population and setting

This was a cross-sectional questionnaire-based study conducted amongst women and gay and other men who have sex with men (MSM) attending the Melbourne Sexual Health Centre (MSHC) in Victoria, Australia, between the 22^nd^ December 2017 and the 22^nd^ February 2018. MSHC is the largest public sexual health service in Victoria, Australia. The centre provides around 50,000 consultations every year, 37% with women and 36% with MSM [12]. Clinic attendees routinely complete a computer assisted self-interview (CASI) about their sexual history prior to seeing a triage nurse.

### Study measurement

Women and MSM presenting to MSHC, aged 18 or over, were invited to complete an electronic questionnaire containing questions about stealthing after completing CASI. Participants read a patient information and consent form which detailed the nature of the survey, and patients could only commence the questionnaire after ticking a box stating ‘Yes- willing to help’. Due to the potential of the questionnaire to cause distress when recalling the stealthing event, the participant information included advertisement of free counselling services available at MSHC and elsewhere. The Alfred Hospital Ethics Committee approved the study (number 494/17).

**Fig 1:**
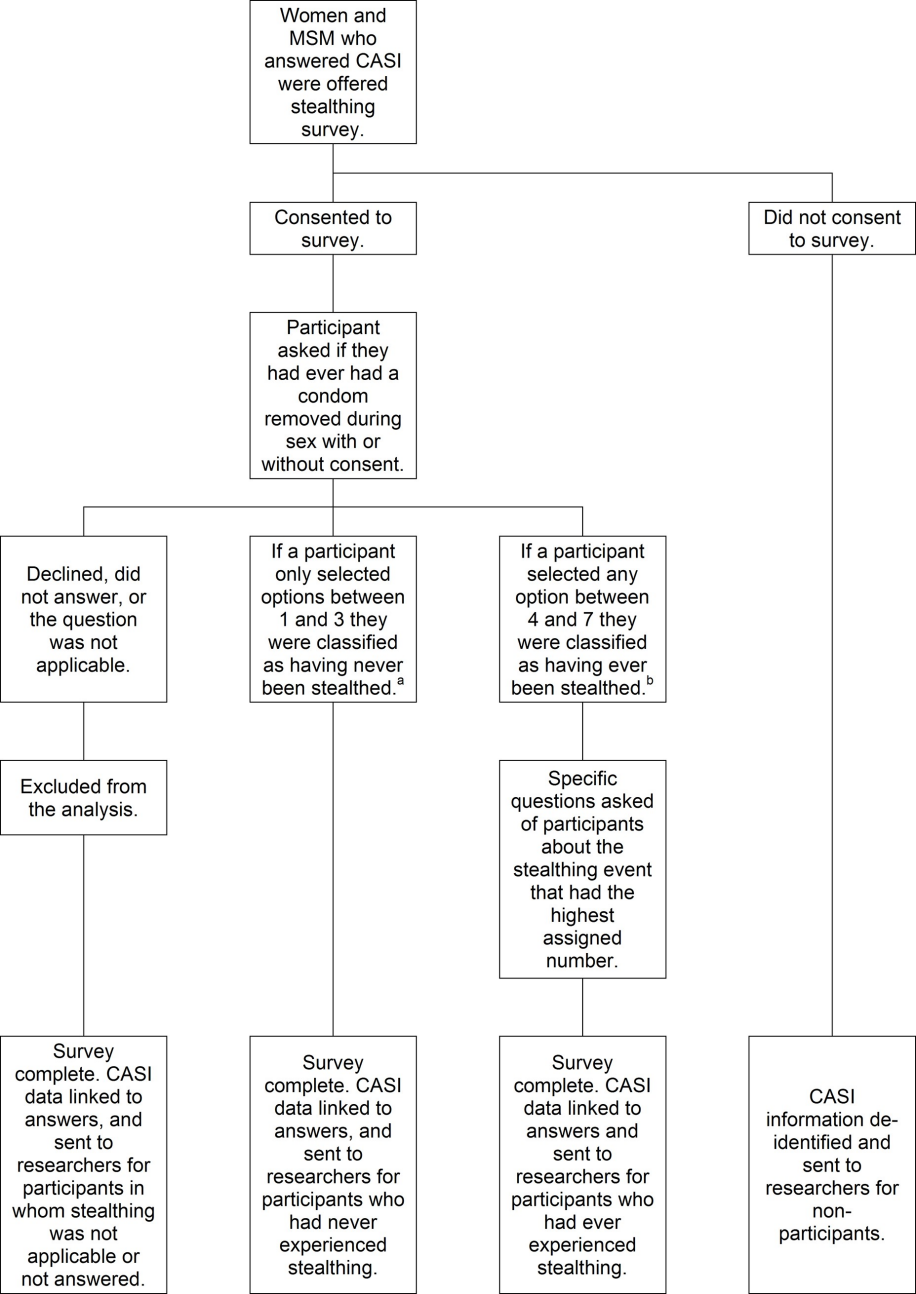
Possible pathways for patients offered the survey, and the classification for analysis of nonconsensual condom removal. Abbreviations: MSM = men who have sex with men; CASI = computer assisted self-interviewing. ^a^Participants were classified as never having experienced stealthing if they responded either: 1) they had never had a condom removed during sex, 2) that a condom had been removed with permission, or 3) that a condom was removed without permission but they willingly continued sex. ^b^Participants were deemed to have experienced stealthing if they reported: 4) condom removal without permission and sex continued unwillingly, 5) condom removal without permission and sex was discontinued, 6) condom removal during sex but they did not realise until afterwards, or 7) the condom was never put on despite being requested.

Age, number of sexual partners, and HIV status were extracted electronically from routinely collected clinic records for respondents and non-respondents, de-identified for non-respondents, and linked to questionnaire responses for respondents (Fig 1).

The questionnaire asked whether the participant had ever had a condom removed during sex with or without permission and at what point the participant noticed. Participants could choose from a hierarchy of seven responses describing the circumstances. Multiple responses were allowed for those reporting multiple occurrences, and there was no time limit applied to the reported event. Participants were deemed not to have experienced stealthing if they responded either: 1) they had never had a condom removed during sex, 2) that a condom had been removed with permission, or 3) that a condom was removed without permission but they willingly continued sex. Participants were deemed to have experienced stealthing if they reported: 4) condom removal without permission and sex continued unwillingly, 5) condom removal without permission and sex was discontinued, 6) condom removal during sex but they did not realise until afterwards, or 7) the condom was never put on despite being requested. If a participant only selected options between 1 and 3 they were classified as never having been stealthed. If a participant selected any option between 4–7, regardless of whether they had also selected options between 1 and 3, they were classified as ever having been stealthed (Fig 1).

Participants who reported stealthing were asked further questions about the specific event (Fig 1). Participants who had selected multiple options were asked about the incident with the highest assigned number. For instance if they reported several stealthing events with differing scenarios and selected both response 4 and 5, then specific questions were asked about “event 5” only–i.e. condom removal without permission and sex was discontinued. Questions included: when the incident occurred, how long they had known the partner, how they would describe the relationship, where they had met, whether either person had been using drugs or alcohol, whether the event was reported to the police, and what they perceived were the consequences of the condom removal. All respondents were asked whether they considered the removal of a condom without consent to be sexual assault.

### Statistical analysis

All analyses were performed using Stata IC version 14. MSM who reported only insertive anal sex and no receptive anal sex while completing CASI were excluded from the dataset prior to analysis of questionnaire responses, as experiencing stealthing was considered unlikely if the male was only the insertive partner. Risk factors for experiencing stealthing in women and MSM were not compared to each other as they are different populations. Univariable and multivariate analyses were performed to determine the differences in demographics between non-respondents and respondents, and the differences between those who had and had not experienced stealthing. Variables were included in multivariate models if the p-value was ≤0.1; if correlated, the variable most strongly associated with the outcome was used. Models were built in a backward-stepwise fashion, using the likelihood ratio test to determine the significance of the contribution of each variable. Ninety-five percent binomial confidence intervals (CIs) were calculated for all proportions. We assumed 100 patients would complete the survey each week and estimated 2% would report ever being stealthed. The 95% confidence interval around an estimated 2% prevalence of stealthing after six weeks (600 responses) would be 1.0%, 3.5%.

## Results

During the study period, 2883 women and 3439 MSM attended the clinic, of whom 1189 women (41%, 95%CI: 39%,43%) and 1063 MSM (31%, 95%CI: 29%,32%) completed the survey (classified as respondents).

Female respondents were more likely than non-respondents to have had sex overseas in the last twelve months (adjusted odds ratio [AOR] 1.49, 95% CI: 1.26,1.77, p<0.001) and were less likely to be a current sex worker (AOR 0.78, 95% CI: 0.63,0.96, p = 0.02) (Table 1). Compared to MSM non-respondents, the men who responded were more likely to have had sex overseas in the last twelve months (AOR 1.70, 95% CI: 1.37,2.11, p<0.001), and were less likely to be HIV positive (AOR 0.60, 95% CI: 0.38,0.95, p = 0.029) (Table 1).

**Table 1:** Demographics and epidemiological features of respondents versus non-respondents to survey on rates of non-consensual removal of condoms (stealthing) in a STI clinic (N = 6322).

	** **	**Female non-respondents n = 1694 (%; 95% CI) or median [range]**		**Female respondents n = 1189 (%; 95% CI) or median [range]**		**Unadjusted Odds Ratio (95% CI)**		**p-value**	**Adjusted Odds Ratio**^**a**^ **(95% CI)**		**p-value**
**Age**		27	[16–74]	26	[18–64]						
**Employment**											
	Employed	958	(60; 57,62)	689	(60; 57,62)	1					
	Not in the labour force^b^	641	(40; 38,43)	467	(40; 38,43)	1.01	(0.87,1.18)	0.87			
**Aboriginal and/or Torres Strait Islander peoples**											
	No	1479	(99; 98,99)	1074	(99; 98,99)	1					
	Yes	16	(1; 0,2)	14	(1; 1,2)	1.20	(0.59,2.48)	0.613			
**Sex overseas**											
	No	817	(60; 57,62)	485	(48; 45,52)	1			1		
	Yes	552	(40; 38,43)	517	(52; 48,55)	1.58	(1.35,1.86)	<0.001	1.49	(1.26,1.77)	<0.001
**Injecting drug use**											
	Never injected	1420	(98; 97,99)	1023	(98; 97,99)	1					
	Ever injected	26	(2; 1,3)	22	(2; 1,3)	1.17	(0.66,2.08)	0.582			
**Current sex worker**											
	No	1095	(76; 74,78)	856	(82; 79,84)	1			1		
	Yes	348	(24; 22,26)	191	(18; 16,21)	0.70	(0.58,0.86)	<0.001	0.78	(0.63,0.96)	0.020
**Condom Use in the last 3mo with male partners**											
	Not always	1014	(83; 81,85)	765	(82; 80,85)	1					
	Always	204	(17; 15,19)	163	(18; 15,20)	1.06	(0.84,1.33)	0.619			
**Number of male sexual partners in the last 3mo**		1	[0–50]	1	[0–15]						
	** **	**Male non-respondents n = 2376 (%; 95% CI) or median [range]**		**Male respondents n = 1063 (%; 95% CI) or median [range]**		**Unadjusted Odds Ratio (95% CI)**		**p-value**	**Adjusted Odds Ratio**^**c**^ **(95% CI)**		**p-value**
**Age**		30	[16–82]	30	[18–75]						
**Employment**											
	Employed	1480	(67; 65,69)	644	(64; 61,67)	1					
	Not in the labour force^b^	742	(33; 31,35)	361	(36; 33,39)	1.12	(0.96,1.31)	0.161			
**Aboriginal and/or Torres Strait Islander peoples**											
	No	2114	(99; 98,99)	978	(99; 99,100)	1			1		
	Yes	26	(1; 1,2)	5	(1; 0,1)	0.42	(0.16,1.09)	0.073	0.64	(0.21,1.97)	0.441
**Sex overseas**											
	No	1365	(70; 69,72)	542	(61; 58,64)	1			1		
	Yes	587	(30; 28,32)	345	(39; 36,42)	1.48	(1.25,1.75)	<0.001	1.70	(1.37,2.11)	<0.001
**Injecting drug use**											
	Never injected	2048	(96; 96,97)	914	(97; 96,98)	1					
	Ever injected	75	(4; 3,4)	28	(3; 2,4)	0.84	(0.55,1.3)	0.428			
**Current sex worker**											
	No	2126	(>99; 99,100)	933	(99; 98,99)	1			1		
	Yes	9	(<1; 0,1)	10	(1; 1,2)	2.53	(1.03,6.25)	0.044	2.72	(0.97,7.59)	0.057
**Condom Use in the last 3mo with male partners**											
	Not always	1379	(74; 72,76)	616	(71; 68,74)	1					
	Always	492	(26; 24,29)	246	(29; 26,32)	1.12	(0.93,1.34)	0.220			
**HIV status**											
	Negative	1279	(91; 90,93)	558	(95; 92,96)	1			1		
	Positive	119	(9; 7,10)	32	(5; 4,8)	0.62	(0.41,0.92)	0.019	0.61	(0.38,0.97)	0.038
**Use of prep**											
	No	1844	(81; 79,82)	861	(83; 81,85)	1					
	Yes	436	(19; 18,21)	174	(17; 15,19)	0.85	(0.70,1.04)	0.112			
**Number of male sexual partners in the last 3mo**		3	[0–100]	3	[0–140]						

Abbreviations: n = number; CI = confidence interval; mo = months; HIV = human immunodeficiency virus; PrEP = HIV pre-exposure prophylaxis

^a^Adjusted model for females includes: sex overseas and current sex worker

^b^Not in the labour force includes both those who are unemployed and/or students

^c^Adjusted model for males includes: Aboriginal and/or Torres Strait Islander peoples, sex overseas, current sex worker and HIV status.

Data missing for: <5% of PrEP data; <5%-10% of employment data; 5–15% of Aboriginal and/or Torres Strait Islander peoples data; 10–15% of current sexworker data; 10%-20% of sex overseas data and injecting drug use data; 15- ≥20% of condom use data; and >20% of HIV data. Proportions are calculated using available data.

Of the 1189 women and 1063 MSM who consented to the survey and answered the first question: 60 (5%) women and 64 men (6%) declined to answer whether they had experienced stealthing, 45 (4%) women and 37 (3%) men deemed the question to be not applicable to them i.e. they never used condoms, or did not engage in penetrative sex with men and 90 (8%) men were removed from the analysis, as they had only reported insertive anal sex and not reported receptive anal sex in CASI (Table 2).

**Table 2:** Reported events of non-consensual removal of condoms (stealthing) amongst patients presenting to a STI clinic (N = 2252)^a^.

		Female respondents n = 1189 (%; 95% CI)		Male respondents n = 1063 (%; 95% CI)	
**Classified as not experiencing ‘stealthing’**					
	Never stealthed	420	(35; 33,38)	496	(47; 44,50)
	Condom removed w permission	455	(38; 35,41)	315	(30; 27,32)
	Condom removed w/o permission but continued willingly	104	(9; 7,10)	77	(7; 6,9)
**Classified as experiencing ‘stealthing’**					
	Condom removed w/o permission, and continued unwillingly	108	(9; 8,11)	52	(5; 4,6)
	Condom removed w/o permission, and stopped	135	(11; 10,13)	65	(6; 5,8)
	Condom removed w/o permission, but didn't realise until afterwards	147	(12; 11,14)	60	(6; 4,7)
	Condom never put on but had been requested	84	(7; 6,9)	41	(4; 3,5)
**Removed from further analysis**					
	Not applicable^b^	45	(4; 3,5)	127	(12; 10,14)
	*Decline answer*	*60*	*(5; 4*,*6)*	*64*	*(6; 5*,*8)*

Abbreviations: n = number; CI = confidence interval; w = with; w/o = without.

^a^Patients could select multiple options, to report multiple events occurring, i.e. events are not mutually exclusive, therefore percentages do not sum to 100. Percentages represent the proportion of participants who have reported the event. If reporting multiple events, patients were classified in the analysis based off the highest numbered event they reported, if 1 is Never and 7 is ‘Condom never put on even though requested’.

^b^Not applicable refers to patients who have not/do not engaged in penetrative penile sex, includes 97 MSM who responded to survey but reported no receptive anal sex and 30 who selected not applicable.

Three hundred and forty-six of the remaining 1084 women (32%, 95% CI: 29%,35%) and 168 of the remaining 872 MSM (19%, 95% CI: 17%,22%) reported having ever experienced stealthing (Table 3). Of those who had experienced stealthing, forty-two women (12%, 95% CI: 9%,16%) and 23 MSM (14%, 95% CI: 9%,20%) presented to the clinic on the day of the questionnaire following a reported stealthing incident (Table 4).

**Table 3:** Risk factors associated with non-consensual removal of condoms (stealthing) in patients presenting to a STI clinic (N = 2042).

		Womenwho have not had been stealthed n = 738 (%; 95% CI) or median [range]		Women who have have been stealthed n = 346 (%; 95% CI) or median [range]		Unadjusted Odds Ratio (95% CI)		p-value	Adjusted Odds Ratio (95% CI)^a^		p-value
**Age**		26	[18–58]	26	[18–55]						
**Number of male sexual partners in the last 3mo**		2	[0–15]	1	[0–15]						
**Employment**^**b**^											
	Employed	439	(61; 57,65)	189	(56; 51,62)	1					
	Not in the labour force	281	(39; 35,43)	146	(44; 38,49)	1.21	(0.93,1.57)	0.161			
**Education level**											
	Did not complete high school	18	(2; 1,4)	13	(4; 2,6)	1					
	High school/Certificate/Diploma	238	(33; 29,36)	134	(39; 34,45)	0.78	(0.37,1.64)	0.512			
	University degree	475	(65; 61,68)	195	(57; 52,62)	0.57	(0.35,1.47)	0.131			
**Aboriginal and/or Torres Strait Islander peoples**											
	No	672	(99; 98,99)	319	(98; 96,99)	1					
	Yes	8	(1; 1,2)	5	(2; 1,4)	1.31	(0.43,4.06)	0.632			
**Australian/New Zealander**											
	No	441	(63; 59,66)	166	(51; 45,56)	1					
	Yes	264	(37; 34,41)	160	(49; 44,55)	1.61	(1.23,2.09)	<0.001	1.26	(0.94,1.70)	0.122
**Current sex worker**											
	No	573	(87; 85,90)	215	(71; 65,76)	1					
	Yes	83	(13; 10,15)	89	(29; 24,35)	2.86	(2.04,4.01)	<0.001	2.87	(2.01,4.11)	<0.001
**Injecting drug use**											
	Never injected	644	(98; 97,99)	295	(97; 95,99)	1					
	Ever injected	11	(2; 1,3)	8	(3; 1,5)	1.59	(0.63,3.99)	0.325			
**Sex overseas**											
	No	303	(47; 44,51)	136	(47; 41,53)	1					
	Yes	335	(53; 49,56)	153	(53; 47,59)	1.02	(0.77,1.34)	0.903			
**Use other contraceptives in addition to condoms**^**c**^											
	No	293	(46; 42,50)	112	(47; 40,53)	1					
	Yes	339	(54; 50,58)	128	(53; 47,60)	0.94	(0.733,1.33)	0.936			
** **	** **	**MSM who have not been stealthed n = 704 (%; 95% CI) or median [range]**		**MSM who have been stealthed n = 168 (%, 95% CI) or median [range]**		**Unadjusted Odds Ratio (95% CI)**		**p-value**	**Adjusted Odds Ratio (95% CI)**^**d**^		**p-value**
**Age**		30	[18–75]	29	[18–58]						
**Number of male sexual partners in the last 3mo**		3	[0–140]	3	[0–100]						
**Employment**											
	Employed	435	(65; 61,69)	98	(61; 53,68)	1					
	Not in the labour force	232	(35; 31,39)	63	(39; 32,47)	1.20	(0.85,1.72)	0.302			
**Education level**											
	Did not complete high school	24	(3; 2,5)	7	(4; 2,8)	1					
	High school/Certificate/Diploma	183	(26; 23,30)	34	(20; 14,27)	0.64	(0.25,1.60)	0.336			
	University degree	494	(70; 67,74)	127	(76; 68,82)	0.88	(0.37,2.09)	0.775			
**Aboriginal and/or Torres Strait Islander peoples**											
	No	701	(100; 99,100)	166	(99; 97,100)	1					
	Yes	2	(0; 0,1)	1	(1; 0,3)	2.11	(0.19,23.42)	0.543			
**Australian/New Zealander**											
	No	343	(50; 46,54)	83	(49; 42,57)	1					
	Yes	342	(50; 46,54)	85	(51; 43,58)	1.03	(0.73,1.44)	0.877			
**Current sex worker**											
	No	622	(99; 98,100)	151	(99; 95,100)	1					
	Yes	5	(1; 0,2)	2	(1; 0,5)	1.65	(0.32,8.57)	0.553			
**Injecting drug use**											
	Never injected	611	(97; 96,98)	145	(96; 92,99)	1					
	Ever injected	18	(3; 2,4)	6	(4; 1,8)	1.4	(0.55,3.60)	0.480			
**Sex overseas**											
	No	354	(60; 56,64)	82	(57; 49,66)	1					
	Yes	237	(40; 36,44)	61	(43; 34,51)	1.11	(0.77,1.61)	0.577			
**HIV status**											
	No	375	(95; 93,97)	96	(2; 85,97)	1					
	Yes	19	(5; 3,7)	8	(8; 3,15)	1.64	(0.70,3.87)	0.255			
**Use of prep**											
	No	582	(84; 81,87)	126	(78; 71,84)	1			1		
	Yes	110	(16; 13,19)	35	(22; 16,29)	1.47	(0.96,2.25)	0.077	1.16	(0.70,1.92)	0.567
**Drugs use with anal sex w/o a condom in the last 12mo**^**e**^											
	No	222	(58; 53,63)	58	(55; 45,64)	1					
	Yes	162	(42; 37,47)	48	(45; 36,55)	1.13	(0.74,1.75)	0.569			
**Drunk during anal sex w/o a condom in the last 12mo**^**e**^											
	No	219	(57; 52,62)	53	(50; 41,60)	1					
	Yes	166	(43; 38,48)	52	(50; 40,59)	1.29	(0.84,1.99)	0.242			
**Anal sex w/o a condom with known HIV positive in the last 12mo**^**e**^											
	No	319	(83; 79,87)	83	(82; 73,89)	1					
	Yes	65	(17; 13,21)	18	(18; 11,27)	1.06	(0.60,1.89)	0.832			
**Anal sex w/o a condom with someone of unknown HIV status in the last 12mo**^**e**^											
	No	189	(50; 45,55)	39	(38; 29,48)	1			1		
	Yes	190	(50; 45,55)	63	(62; 52,71)	1.61	(1.03,2.51)	0.038	1.51	(0.96,2.39)	0.075
**Self-reported health issues, such as anxiety or depression, which may have affected your decision to use condoms for anal sex?**^**e**^											
	No	318	(85; 81,89)	74	(73; 63,81)	1			1		
	Yes	55	(15; 11,19)	28	(27; 19,37)	2.19	(1.30,3.68)	0.003	2.13	(1.25,3.6)	0.005

Abbreviations: n = number; CI = confidence interval; mo = months; MSM = men who have sex with men; HIV = human immunodeficiency virus; PrEP = HIV pre-exposure prophylaxis; w/o = without

^a^Adjusted model for females includes: Australian and current sex worker

^b^Not in the labour force includes both those who are unemployed and/or students

^c^Women who reported not using contraception due to pregnancy were excluded (2 females who did not have condoms removed, and 10 who did).

^d^Adjusted model for males includes: use of prep, condom use with someone of uncertain HIV status, health issues (anxiety & depression) affecting decisions to use condoms.

^e^These questions were asked only to patients who had reported unprotected anal sex since their last HIV test as part of their routine computer assisted self-interviewing (CASI).

Data missing for: <5% of employment data, education data and PrEP data; <5%-10% of Aboriginal and/or Torres Strait Islander peoples data and Australian data; 10%-15% of injecting drug use data and current sex worker data, 10%-20% sex overseas data; 10- ≥20% contraception data; and ≥20% of HIV status and questions on issues affecting decisions to use condoms. Proportions are calculated using available data.

**Table 4:** Situational factors surrounding non-consensual removal of condoms (stealthing) reported by patients presenting to a STI clinic (N = 523).

	* *	Women n = 346 (%; 95% CI)		MSM n = 168 (%; 95% CI)	
**When the incident occurred**					
	Here today for this reason	42	(12; 9,16)	23	(14; 9,20)
	In the last 3mo	59	(17; 13,22)	20	(12; 7,18)
	3–12 mo ago	78	(23; 18,28)	35	(21; 15,28)
	More than 12 months ago	120	(35; 30,40)	78	(46; 39,54)
	More than 1 occasion	43	(13; 9,17)	12	(7; 4,12)
**Relationship**					
	Did not know him well	101	(30; 25,36)	102	(61; 54,69)
	Friend	33	(10; 7,14)	10	(6; 3,11)
	Friend with benefits/ Sex buddy	51	(15; 12,20)	30	(18;13,25)
	Casually dating	54	(16; 12,21)	22	(13; 8,19)
	Relationship	25	(8; 5,11)	2	(1; 0,4)
	Client (of sex worker)	69	(21; 16,25)	0	(0; 0,2)^a^
**Relationship duration**					
	Less than a day (<24hrs)	126	(38; 33,44)	85	(52; 44,59)
	One day to one month	95	(29; 24,34)	39	(24; 17,31)
	More than one month	107	(33; 28,28)	41	(25; 18,32)
**Met through**					
	Smartphone dating app/Internet	64	(20; 15,24)	110	(67; 59,74)
	(Gay) bar or party	50	(15; 12,20)	20	(12; 8,18)
	Gay sauna, beats of SOPV, sex party	2	(1; 0,2)	24	(15; 10,21)
	Friend, or friend of friend	94	(29; 24,34)	6	(4; 1,8)
	Co-workers	22	(7; 4,10)	3	(2; 0,5)
	Sex work	76	(23; 19,28)	0	(0; 0,2)^a^
	Travel	15	(5; 3,7)	0	(0; 0,2)^a^
	Other (café, park etc.)	4	(1; 0,3)	1	(1; 0,3)
**Drugs used by partner**^**b**^^**c**^					
	None	75	(27; 22,33)	63	(53; 44,62)
	Alcohol	188	(68; 62,73)	48	(40; 31,50)
	Cannabis/marijuana/hash	28	(10; 7,14)	4	(3; 1,8)
	Ecstasy	12	(4; 2,7)	4	(3; 1,8)
	Speed/ice/meth	5	(2; 1,4)	6	(5; 2,11)
	GHB	2	(1; 0,3)	3	(2; 1,7)
	Cocaine	10	(4; 2,7)	3	(2; 1,7)
	Heroin	1	(<1; 0,2)	0	(0; 0,3)^a^
	Other	1	(<1; 0,2)	3	(2; 1,7)
**Drugs used by respondent**^**b**^^**d**^					
	None	135	(41; 36,47)	87	(54; 46,62)
	Alcohol	186	(57; 51,62)	65	(41; 33,49)
	Cannabis/marijuana/hash	21	(6; 4,10)	3	(2; 0,5)
	Ecstasy	9	(3; 1,5)	4	(3; 1,6)
	Speed/ice/meth	5	(2; 0,4)	8	(5; 2,9)
	GHB	2	(1; 0,2)	3	(2; 0,5)
	Cocaine	8	(2; 1,5)	4	(3; 1,6)
	Heroin	1	(<1; 0,2)	0	(0; 0,2)^a^
	Other	0	(0; 0,1)^a^	4	(3; 1,6)
**Condom removal discussed with partner**					
	No	128	(39; 33,44)	74	(45; 37,52)
	Yes	204	(61; 56,67)	92	(55; 48,63)
**Consequences of condom removal**^**b**^					
	None	85	(25; 21,30)	62	(38; 30,46)
	Emotional stress	190	(56; 51,62)	86	(52; 45,60)
	Caught an STI	26	(8; 5,11)	9	(5; 3,10)
	Contracted HIV	2	(1; 0,2)	3	(2; 0,5)
	Fight	49	(14; 11,19)	15	(9; 5,15)
	Relationship broke up	30	(9; 6,12)	6	(4; 1,8)
	Other	42	(12; 9,16)	12	(7; 4,12)
**Reported to the police**					
	No	336	(99; 97,100)	163	(98; 95,100)
	Yes	3	(1; 0,3)	3	(2; 0,5)

Abbreviations: n = number; CI = confidence interval; MSM = men who have sex with men; mo = months; SOPV = sex on premises venue; GHB = Gamma-hydroxybutyrate; STI = sexually transmitted infection; HIV = human immunodeficiency virus

^a^one-sided, 97.5% confidence interval

^b^Patients could select multiple options, to report multiple events occurring, i.e. events are not mutually exclusive, therefore percentages do not sum to 100. Percentages represent the proportion of participants who have reported the event.

^c^64 women (19%) and 47 MSM (28%) were unsure as to whether or not their partner had used any alcohol and/or other drugs and were removed from the analysis.

^d^11 women (3%) and 6 MSM (4%) were unsure as to whether or not they had used any alcohol and/or other drugs and were removed from the analysis.

Data missing from up to 5% of female respondents and up to 3% of male respondents; proportions are calculated using available data.

On multivariate analysis, women who had been stealthed were more likely to be a current sex worker than those who had never experienced stealthing (AOR 2.87, 95% CI: 2.01,4.11, p<0.001) (Table 3), and MSM who had been stealthed were more likely to report ‘health issues, such as anxiety or depression which may have affected their decision to use condoms for anal sex’ than those who had never experienced stealthing (AOR 2.13, 95% CI: 1.25,3.60, p = 0.005) (Table 3).

Most women met the male partner who had stealthed them through friends (29%, 95% CI: 24%,34%) or sex work (23%, 95% CI: 19%,28%). MSM reporting stealthing most commonly described the partner as someone they “did not know well” (61%) and had predominantly met them through geosocial dating applications or online (67%, 95% CI: 59%,74%) (Table 4).

At the time of the stealthing incident, 41% (95% CI: 36%,47%) of women and 54% (95% CI: 46%,62%) of MSM reported being sober, while 57% (95% CI: 51%,62%) of women and 41% (95% CI: 33%,49%) of MSM had consumed alcohol. Twelve percent of women and 13% of MSM had used other drugs either in addition to or without alcohol (Table 4). The majority of women reported their partner had consumed alcohol (68%, 95% CI: 62%,73%) and/or other drugs (19%), with only 27% (95% CI: 22%,33%) stating the partner had been sober when the incident occurred. Many MSM believed their partner to be sober (53%, 95% CI: 44%,62%), with 40% (95% CI: 31%,50%) of partners under the influence of alcohol, and 12% using additional/or other drugs (Table 4).

The majority of women (61%) and MSM (55%) discussed the removal of the condom with their partners after the event. Over half of the participants reported being emotionally stressed following the incident. Eight percent of women and five percent of MSM reported they thought they had acquired an STI following the event. One percent of women and two percent of MSM believed they had acquired HIV as a consequence of being stealthed (Table 4). Only 1% of people stealthed reported this experience to the police (Table 4).

Both female and MSM participants who had experienced stealthing were less likely to consider it to be sexual assault than participants who had not experienced stealthing. Amongst women, 62% (95% CI: 56%,67%) of those stealthed considered it to be assault, compared to 85% (95% CI: 82%,87%) of those not stealthed (OR 0.29, 95%CI: 0.22,0.4, p<0.001). Amongst men, 61% (95% CI: 53%,69%) of those stealthed considered it to be assault versus 84% (95% CI: 81%,86%) of those not stealthed (OR 0.31, 95%CI: 0.21,0.45, p<0.001).

## Discussion

Although increasingly discussed in international media, there is little scientific research on non-consensual removal of condoms, popularly termed ‘stealthing’. To our knowledge this is the first study investigating how common stealthing is, the context in which it occurred, the impact on individuals, and how those stealthed perceive the event. A surprising proportion of clients attending a sexual health centre in Melbourne (32% of women and 19% of MSM) reported removal of a condom in a situation where they would not have willingly engaged in sexual intercourse without one—in other words, a violation of their consent [1].

These data need to be interpreted in the context of a STI clinic population which is generally a higher risk group than the general population. Our data show that 4% of women and 3% of MSM presenting to our clinic during the study period were attending following a stealthing incident. This equates to over 1200 consultations per year [12]. These data suggest that stealthing is common and should be considered when assessing patients in STI services.

Female respondents were less likely to be a current sex worker and MSM respondents were less likely to be HIV positive, compared to non-respondents. It is possible that both sex workers and HIV positive men were less likely to complete the survey due to privacy concerns, especially with regards to condom use and their legal obligations, which vary state by state in Australia. In Victoria, sex workers are legally required to use condoms with clients[13], and while those who are HIV positive are not legally required to disclose their HIV status, they must take reasonable precautions to prevent HIV transmission to those they are engaging in penetrative sex with[14]. Reasonable precaution refers to correct use of condoms and lube during intercourse. While female sex workers were less represented in respondents than non-respondents, 18% of participants were sex workers and we still observed an association between being a sex worker and being more likely to be stealthed. Low numbers of HIV positive men participating may have limited our ability to examine any association between stealthing and HIV status. Lastly, both women and MSM who had been overseas recently were more likely to respond to our survey. This may bias our findings towards individuals who may have participated due to recent high risk sexual encounters, in the context of overseas travel[15].

Women who experienced stealthing were three times more likely to be sex workers compared to those who had not. In the Law and Sex Worker Health (LASH) Survey conducted in Australia, 8% of respondents reported assault by clients [16]. However the LASH survey did not compare rates of assault to the general population or differentiate between physical and sexual assault, and only examined assault in the workplace. Perkins' (1991) research with Sydney-based brothel workers found that 20% of sex workers experienced rape while working. Outside the workplace sex workers experienced higher levels of sexual assault compared with non-sex workers, with 46.9% reporting rape, compared to 21.9% of health workers and 12.7% of students [17]. Our data are consistent with these findings that sex workers are at increased risk of non-consensual sex acts.

Sixty-seven percent of MSM who had experienced stealthing met the partner via geosocial dating applications, for example Grindr, Tinder or Scruff. This is comparable to the number of MSM meeting partners through dating applications (70%) [18]. Sexual encounters initiated online are more likely to include unprotected anal intercourse [19], however it has also been found that meeting partners online increases the likelihood of discussion between partners of preferred sexual practices compared to meeting partners offline [19, 20]. MSM who had been stealthed were twice as likely to report having anxiety or depression. Depressive symptoms and anxiety are predictive of condom non-use [21] and higher levels of depression are related to lower levels of self-efficacy for sexual safety [22]. MSM who have anxiety or depression may be vulnerable to stealthing for this reason.

In this study, the majority of women (73%) believed the partner who had stealthed them to be under the influence of alcohol and/or other drugs. In heterosexual relations, the link between alcohol consumption and committal of sexual assault is well documented [23, 24]. Condom resistance tactics and sexual aggression with female partners are more commonly employed by men with history of sexual aggression and alcohol intoxication [25, 26]. Additionally, both alcohol consumption [27] and condom use [28, 29] have been associated with erectile dysfunction. Men with erection issues are more likely to engage in unprotected sex, misuse condoms [28, 29], and are more likely to remove condoms before sex is over (p = 0.001) [29]. Literature supports our finding that heterosexual men who have consumed alcohol may be at increased risk of committing nonconsensual sex acts, and may be removing the condom to maintain an erection.

Whilst the majority of those reporting stealthing considered it sexual assault, they were three times less likely to consider stealthing sexual assault than those who had never experienced it. The US National Crime Victimization Survey found 20% of female victim narratives contained excuses for offenders’ behaviour, denials of injury, or justification of the incident as the victims’ fault [30]. This allowed the women to avoid the distress of labelling themselves victims of a crime, or their partners as criminals [30]. Victims of stealthing may also not yet view themselves as sexual assault victims as stealthing is a relatively new topic. Sexual assault is a term with many connotations and there are cultural myths as to who is a ‘real’ sexual victim [31], with the type of violence experienced influencing society’s view as to whether a woman is a victim [32]. Our current language around sexual assault (and in this case, stealthing) may require expansion- until an act is named as assault it cannot be viewed as such, and cannot be reported or legislated against [33]. A limitation of this study is that we did not ask respondents why they did not consider stealthing to be sexual assault.

Stealthing has potentially serious consequences. The majority of patients reported consequences following the stealthing incident, with over half experiencing emotional stress. Although literature contains estimates as to the rate of STI and HIV transmission during sexual assault, it is difficult to establish if an STI has been acquired from a specific event. The Centers for Disease Control and Prevention (CDC) guidelines recommend testing all people for STIs following sexual assault [34], with the caveat that many positive tests will be from a pre-existing STI [35]. MSM patients with condom malfunction or condom-less sex presenting in a 72 hour window fulfil criteria for HIV Non-Occupational Post Exposure Prophylaxis (nPEP) [36, 37], and therefore MSM who present reporting non-consensual condom removal should be prescribed it.

This study has several limitations. Firstly, this study was offered in English only, which means it cannot be generalised to attendees who are not fluent in English. Secondly, this study may be subject to responder bias, as those who have experienced stealthing may have been more likely to answer the survey. Given this is a retrospective survey, participant responses may be subject to recall bias, and specific contextual situational factors and outcomes were asked about one event only for those stating it had happened on more than one occasion. While some participants within our study attributed the acquisition of STIs to being stealthed, this cannot be verified. According to attribution theory [38] following an adverse event people will make attributions to understand and control their environment[39], with situational factors often exaggerated when there is a negative outcome [40], and thus patients could be incorrectly attributing contracting a STI to the stealthing event.

Despite these limitations, this study has a large sample size with over two thousand responses. Accurate statistics describing the prevalence and incidence of sexual assault are difficult to obtain since the majority of assaults are not reported to authorities and victims often do not access services [31]. Only 1% of patients reporting stealthing in this study reported the event to the police. Although this study may be subject to recall bias, population surveys are the best means of learning the true extent and nature of these crimes, rather than relying on crime statistics. This is the first study to describe how commonly this practice is occurring.

In summary, stealthing was commonly experienced by our clinic population, with a third of women and a fifth of MSM reporting it, with situational contexts often involving alcohol and/or drugs in women, and geosocial networking applications in MSM. Sex work was a clear risk factor identified among women, and risk factors for MSM included anxiety and depression. Knowledge of these risk factors can enable services to ask about stealthing in target groups and offer specific support and counselling. Further community-based research would help determine the prevalence in the broader population and studies that link behavioural measures to biological outcomes would help to quantify the STI risk associated with this practice.
