# Correction: Non-consensual condom removal, reported by patients at a sexual health clinic in Melbourne, Australia

**DOI:** 10.1371/journal.pone.0213316

**Published:** 2019-02-28

**Authors:** The PLOS ONE Staff

There is an error in the caption for Table 4, “Situational factors surrounding non-consensual removal of condoms (stealthing) reported by patients presenting to a STI clinic (N = 523).” The publisher apologizes for the error. Please see the complete, correct Table 4 here.

**Table 4:** Situational factors surrounding non-consensual removal of condoms (stealthing) reported by patients presenting to a STI clinic (N = 523).

	Women n = 346 (%; 95% CI)		MSM n = 168 (%; 95% CI)	
**When the incident occurred**				
Here today for this reason	42	(12; 9,16)	23	(14; 9,20)
In the last 3mo	59	(17; 13,22)	20	(12; 7,18)
3–12 mo ago	78	(23; 18,28)	35	(21; 15,28)
More than 12 months ago	120	(35; 30,40)	78	(46; 39,54)
More than 1 occasion	43	(13; 9,17)	12	(7; 4,12)
**Relationship**				
Did not know him well	101	(30; 25,36)	102	(61; 54,69)
Friend	33	(10; 7,14)	10	(6; 3,11)
Friend with benefits/ Sex buddy	51	(15; 12,20)	30	(18; 13,25)
Casually dating	54	(16; 12,21)	22	(13; 8,19)
Relationship	25	(8; 5,11)	2	(1; 0,4)
Client (of sex worker)	69	(21; 16,25)	0	(0; 0,2)^a^
**Relationship duration**				
Less than a day (<24hrs)	126	(38; 33,44)	85	(52; 44,59)
One day to one month	95	(29; 24,34)	39	(24; 17,31)
More than one month	107	(33; 28,28)	41	(25; 18,32)
**Met through**				
Smartphone dating app/Internet	64	(20; 15,24)	110	(67; 59,74)
(Gay) bar or party	50	(15; 12,20)	20	(12; 8,18)
Gay sauna, beats of SOPV, sex party	2	(1; 0,2)	24	(15; 10,21)
Friend, or friend of friend	94	(29; 24,34)	6	(4; 1,8)
Co-workers	22	(7; 4,10)	3	(2; 0,5)
Sex work	76	(23; 19,28)	0	(0; 0,2)^a^
Travel	15	(5; 3,7)	0	(0; 0,2)^a^
Other (café, park etc.)	4	(1; 0,3)	1	(1; 0,3)
**Drugs used by partner**^b^^c^				
None	75	(27; 22,33)	63	(53; 44,62)
Alcohol	188	(68; 62,73)	48	(40; 31,50)
Cannabis/marijuana/hash	28	(10; 7,14)	4	(3; 1,8)
Ecstasy	12	(4; 2,7)	4	(3; 1,8)
Speed/ice/meth	5	(2; 1,4)	6	(5; 2,11)
GHB	2	(1; 0,3)	3	(2; 1,7)
Cocaine	10	(4; 2,7)	3	(2; 1,7)
Heroin	1	(<1; 0,2)	0	(0; 0,3)^a^
Other	1	(<1; 0,2)	3	(2; 1,7)
**Drugs used by respondent**^b^^d^				
None	135	(41; 36,47)	87	(54; 46,62)
Alcohol	186	(57; 51,62)	65	(41; 33,49)
Cannabis/marijuana/hash	21	(6; 4,10)	3	(2; 0,5)
Ecstasy	9	(3; 1,5)	4	(3; 1,6)
Speed/ice/meth	5	(2; 0,4)	8	(5; 2,9)
GHB	2	(1; 0,2)	3	(2; 0,5)
Cocaine	8	(2; 1,5)	4	(3; 1,6)
Heroin	1	(<1; 0,2)	0	(0; 0,2)^a^
Other	0	(0; 0,1)^a^	4	(3; 1,6)
**Condom removal discussed with partner**				
No	128	(39; 33,44)	74	(45; 37,52)
Yes	204	(61; 56,67)	92	(55; 48,63)
**Consequences of condom removal**^b^				
None	85	(25; 21,30)	62	(38; 30,46)
Emotional stress	190	(56; 51,62)	86	(52; 45,60)
Caught an STI	26	(8; 5,11)	9	(5; 3,10)
Contracted HIV	2	(1; 0,2)	3	(2; 0,5)
Fight	49	(14; 11,19)	15	(9; 5,15)
Relationship broke up	30	(9; 6,12)	6	(4; 1,8)
Other	42	(12; 9,16)	12	(7; 4,12)
**Reported to the police**				
No	336	(99; 97,100)	163	(98; 95,100)
Yes	3	(1; 0,3)	3	(2; 0,5)

Notes:

Abbreviations: n = number; CI = confidence interval; MSM = men who have sex with men; mo = months; SOPV = sex on premises venue; GHB = Gamma-hydroxybutyrate; STI = sexually transmitted infection; HIV = human immunodeficiency virus

^a^one-sided, 97.5% confidence interval

^b^Patients could select multiple options, to report multiple events occurring, i.e. events are not mutually exclusive, therefore percentages do not sum to 100. Percentages represent the proportion of participants who have reported the event.

^c^64 women (19%) and 47 MSM (28%) were unsure as to whether or not their partner had used any alcohol and/or other drugs and were removed from the analysis.

^d^11 women (3%) and 6 MSM (4%) were unsure as to whether or not they had used any alcohol and/or other drugs and were removed from the analysis.

Data missing from up to 5% of female respondents and up to 3% of male respondents; proportions are calculated using available data.
